# Effects of *N*-alkyl quaternary quinuclidines on oxidative stress biomarkers in SH-SY5Y cells

**DOI:** 10.2478/aiht-2025-76-4007

**Published:** 2025-09-30

**Authors:** Antonio Zandona, Lucija Marcelić, Suzana Žunec, Josip Madunić, Maja Katalinić

**Affiliations:** Institute for Medical Research and Occupational Health, Division of Toxicology, Zagreb, Croatia

**Keywords:** DNA damage, glutathione, mitochondrial dysfunction, oximes, reactive nitrogen species, reactive oxygen species, superoxide dismutase, glutation, mitohondrijska disfunkcija, oksimi, oštećenje DNA, reaktivne dušikove vrste, reaktivne kisikove vrste, superoksid-dismutaza

## Abstract

Having a broad spectrum of biological and pharmacological activities, including anticholinergic, antihistaminic, antiparasitic, antioxidant, and antitumour, quinuclidine derivatives have piqued much interest in the field of drug discovery and biomedical research. This study investigates the oxidative stress effects of six *N*-alkyl quaternary quinuclidine derivatives, namely three oximes (QNOH-C_12,14,16_) and three alcohols (QOH-C_12,14,16_) in SH-SY5Y neuroblastoma cells to evaluate their safety profile as potential therapeutics. We treated SH-SY5Y cells with their lowest-observed-adverse-effect levels (LOAEL) for 4 h and measured reactive oxygen/nitrogen species (ROS/RNS), lipid peroxidation, glutathione (GSH), superoxide dismutase (SOD) activity, and DNA damage. Q(N)OHs significantly increased ROS and RNS levels, particularly the QNOHs, while lipid peroxidation remained unaffected, and GSH depletion was minimal. Cytosolic Cu/Zn-SOD activity increased significantly with longer alkyl chain substituents, while Mn-SOD activity decreased, indicating mitochondrial damage. DNA damage was not elevated. The observed effects of the tested quinuclidine derivatives appear to depend on their structural properties, as compounds containing hydroxyl groups and shorter alkyl chains had a lower impact. Furthermore, even non-cytotoxic doses of the tested compounds affected cell homeostasis, which underlines the importance of such testing early in the evaluation of new potential drugs.

## Abstract (hr)

Zbog širokog spektra bioloških i farmakoloških aktivnosti, uključujući antikolinergičko, antihistaminsko, antiparazitsko, antioksidacijsko i antitumorsko djelovanje, derivati kinuklidina izazvali su velik interes u području razvoja novih lijekova i biomedicinskih istraživanja. U ovom su istraživanju analizirani učinci oksidacijskog stresa šest *N*-alkil kvaternih derivata kinuklidina – tri oksima (QNOH-C_12,14,16_) i tri alkohola (QOH-C_12,14,16_) – u stanicama neuroblastoma SH-SY5Y kako bi se procijenio njihov sigurnosni profil kao potencijalnih terapeutika. Stanice SH-SY5Y tretirane su najnižim koncentracijama koje prouzročuju štetni učinak (LOAEL) tijekom 4 sata, nakon čega su izmjerene razine reaktivnih kisikovih i dušikovih vrsta (ROS i RNS), peroksidacije lipida, glutationa (GSH), aktivnosti superoksid dismutaze (SOD) i oštećenja DNA. Spojevi Q(N)OH značajno su povećali razine ROS i RNS, osobito QNOH spojevi; peroksidacija lipida nije bila potaknuta, a smanjenje razine GSH bilo je minimalno. Aktivnost citosolnoga Cu/Zn-SOD značajno je rasla s duljinom alkilnoga lanca, dok je aktivnost mitohondrijskoga Mn-SOD opadala, što upućuje na oštećenje mitohondrija. Nije zabilježeno povećanje oštećenja DNA. Uočeni učinci testiranih kinuklidinskih derivata ovise o njihovim strukturnim svojstvima, pri čemu su spojevi s hidroksilnim skupinama i kraćim alkilnim lancima pokazali manji utjecaj. Štoviše, čak su i necitotoksične doze ispitivanih spojeva narušile staničnu homeostazu, što naglašava važnost ovakva testiranja u ranoj fazi procjene sigurnosti novih potencijalnih lijekova.

Quinuclidinium compounds are a diverse class of natural or synthetic chemical agents with a wide range of potential applications in medicine and industry (1,2,3,4,5,6). These compounds are characterised by a rigid bicyclic core (7) that allows specificity, adaptability, and unique electrostatic properties in various chemical interactions, which makes them ideal candidates for binding to biological targets (8, 9). Recent advances in organic synthesis have enabled the development of synthetic quinuclidinium compounds that enhance the properties of their natural analogues with improved potency, stability, and selectivity for specific biological targets, such as receptors and enzymes, including cholinesterases (4, 10,11,12,13,14). From the therapeutic viewpoint, inhibition of acetyl- and butyrylcholinesterase can increase acetylcholine levels and thus improve cognitive function impaired by neurodegenerative diseases (15).

Several quinuclidinium compounds have shown potent cholinesterase inhibition, but also adverse effects like cytotoxicity. One of the intriguing aspects of quinuclidinium compound activity is biological response to the increase in aliphatic side chain length, as it can enhance the binding efficiency and/or cytotoxicity of quinuclidinium compounds (2,3,4, 14, 16). Better understanding of the relationship between the side chain length and biological activity may reveal structural factors that contribute to the therapeutic efficacy and safety of these compounds. Therefore, we focused on six quinuclidinium analogues of different side chain lengths, previously shown to be cytotoxic, to relate their oxidative effects to their structural properties in a SH-SY5Y cell model.

## MATERIALS AND METHODS

### Chemicals and cell culture

The six tested *N*-alkyl quaternary quinuclidine compounds (Q(N)OH-C_12,14,16_) were designed and synthesised by the Professor Ines Primožič group at the University of Zagreb Faculty of Science, Department of Chemistry (Zagreb, Croatia) as described elsewhere (4, 14). Q(N)OHs were prepared as 100 mmol/L stock solutions in dimethyl sulfphoxide (DMSO) and diluted to a designated concentration in unsupplemented cell medium prior to experimentation. The selected concentrations correspond to the lowest observed adverse effect level (LOAEL), that is, the concentration achieving 20 % growth inhibition (IC_20_) at 4 h of exposure, determined in our previous study (14), as follows: 60 µmol/L for QOH-C_12_, 6 µmol/L for QOH-C_14_ and QOH-C_16_, 100 µmol/L for QNOH-C_12_, 25 µmol/L for QNOH-C_14_, and QNOH-C_16_.

The cell line used in the experiments was human neuroblastoma SH-SY5Y obtained from the European Collection of Authenticated Cell Cultures (ECACC, Salisbury, UK; Cat. No. 94030304). Cells were cultivated in Dulbecco’s Modified Eagle Medium (DMEM) F12 with 15 % foetal bovine serum (FBS), 1 % penicillin/streptomycin, and 1 % non-essential amino acids (all from Sigma-Aldrich Chemie, Steinheim, Germany) at 37 °C in a 5 % CO_2_-enriched atmosphere with regular medium changes and passaging as needed.

### Measurement of total antioxidative capacity

Total antioxidative capacity (TAC) in cell lysates was measured using the ferric reducing antioxidant power (FRAP) assay as described elsewhere (17) with slight adjustments to 96-well microplates (18, 19). The working FRAP reagent was prepared by mixing acetate buffer (300 mmol/L, pH 3.6) and a solution of 10 mmol/L tripyridyltriazine (TPTZ) in 40 mmol/L HCl and 20 mmol/L FeCl_3_ in the 10:1:1 (*v*/*v*/*v*) ratio. All the chemicals were purchased from Sigma-Aldrich (St. Louis, MO, USA), except for TPTZ (Fluka, Buchs, Switzerland), and FeCl_3_ (Kemika, Zagreb, Croatia).

Briefly, SH-SY5Y cells were seeded at a density of 400,000 cells/well in 24-well plates. After the 4-hour treatment, we added ice-cold phosphate-buffered saline (PBS) to the wells, harvested the cells with a rubber scrapper, transferred the suspension into microtubes, and sonicated them at 60 % amplitude with a UP50H ultrasonic processor (Hielscher Ultrasonics, Teltow, Germany) for half a cycle. 10 µL of the obtained cell suspension was added to 240 µL of FRAP reagent in a 96-well plate and incubated at 37 °C for 60 min. The absorbance was measured at 593 nm using a SpectraMax iD3 microplate reader (Molecular Devices, San Jose, CA, USA). TACs were calculated from the standard curve obtained from the absorbance of known Fe_2_SO_4_ × 7H_2_O concentrations (0.02–1.0 mmol/L) and are expressed in µmol/L.

### Measurement of reactive oxygen and nitrogen species

Reactive oxygen and nitrogen species (ROS and RNS, respectively) induced by the six Q(N)OHs were determined using one of the following cell-permeable reagents: 2′,7′-dichlorofluorescein diacetate dye (DCFDA) or 4-amino-5-methylamino-2,7-difluorofluorescein diacetate (DAF-FM) (both from Sigma-Aldrich Chemie).

SH-SY5Y cells were seeded at a density of 20,000 cells/well in 96-well black plates. Q(N)OHs were added with either DCFDA or DAF-FM (2 µmol/L final) and pluronic acid (PA) (Sigma-Aldrich Chemie) in the final concentration of 16 µmol/L as described elsewhere in detail (20). The plate was stirred gently and immediately inserted into the plate reader (SpectraMax^®^ iD3, Molecular Devices) and fluorescence registered at 495/529 nm (for DCFDA) or 485/535 nm (for DAF-FM) every 3–10 min for 4 h for ROS or for 1 h for RNS, as described in detail elsewhere (21, 22). For ROS or RNS positive controls we used hydrogen peroxide (H_2_O_2_) or sodium nitroprusside dihydrate (SNP) (both from Sigma-Aldrich Chemie) in the final concentrations of 100 or 200 µmol/L, respectively. Results are expressed as normalised fluorescence signal compared to negative control.

### Measurement of thiobarbituric acid reactive substances

The end products of lipid peroxidation, e.g. malondialdehyde, were measured using the thiobarbituric acid reactive substance (TBARS) following a previously described protocol (22). Briefly, SH-SY5Y cells were seeded at a density of 400,000 cells/well in 24-well plates. After the 4-h treatment, cells were harvested and sonicated as described above. 200 µL of cell suspension was added to 200 µL of thiobarbituric/trichloroacetic acid (TBA/TCA) reagent and heated to 90 °C for 30 min. Then, it was immediately cooled in an ice bath and centrifuged at 1,006 *g* and 4 °C for 5 min (Universal 32R centrifuge, Hettich Instruments, Beverly, MA, USA). Absorbances were measured on SpectraMax iD3 (Molecular Devices) at 532 nm.

TBARS concentrations were calculated using a standard curve constructed with 1,1,3,3-tetrametoxypropane (0.3–6.0 µmol/L) and expressed as µmol/L.

### Glutathione measurement

For glutathione (GSH) measurement we used monochlorobimane (MCB, Sigma-Aldrich Chemie) as fluorescent probe and followed the protocol described elsewhere (23). *Tert*-butyl hydrogen peroxide (tBHP, Sigma-Aldrich Chemie) in the final concentration of 100 µmol/L was used as positive control. After the treatment, fluorescence was recorded on an Infinite M200PRO plate reader (Tecan Austria GmbH, Salzburg, Austria) with excitation and emission spectra of 355/460 nm. Results are expressed as normalised fluorescence signal compared to negative control.

### Superoxide dismutase activity measurement

Superoxide dismutase (SOD) activity was measured using the SOD Assay Kit (Cayman Chemical, Ann Arbor, MI, USA). After the 4-h quinuclidine derivative treatment, the cells were harvested with ice-cold SOD-buffer (20 mmol/L HEPES pH 7.2, 1 mmol/L EGTA, 210 mm mannitol, 70 mmol/L sucrose), sonicated, and centrifuged at 1,500 *g* and 4 °C for 5 min. Supernatant was removed and centrifuged one more time at 10,000 *g* and 4 °C for 15 min. Supernatant was then removed for the assay (Cu/Zn-SOD), while the pellet was resuspended in SOD buffer (Mn-SOD). Samples and standards, 10 µL/well, were mixed in a transparent 96-well plate with 200 µL of diluted radical detector and 20 µL diluted xanthine oxidase from the kit and incubated in a shaker at 22–24 °C for 30 min. Absorbance was recorded on an Infinite M200PRO (Tecan Austria) plate reader at 450 nm. The final concentration of 100 µmol/L of tBHP (Sigma-Aldrich Chemie) was used as positive control.

Results are expressed as SOD activity normalised to protein concentration (U/mg of protein). Protein concentrations were measured using the biochonic acid (BCA) assay (Pierce™ BCA Protein Assay Kit, Thermo Fisher Scientific, Waltham, MA, USA) as described elsewhere (21). Absorbance was detected on a plate reader at 570 nm.

### Measurement of DNA damage

DNA damage was assessed by measuring the activation of ATM kinase and phosphorylated histone H2A.X using the Muse Multi-Color DNA Damage Kit for the Guava Muse Cell Analyzer (Luminex, Częstochowa, Poland). The procedure followed a previously described protocol (24) based on specific antibodies. 24-h treatment with 10 µmol/L etoposide (Sigma-Aldrich Chemie) served as a positive control. The results are presented as percentage of total DNA damage relative to negative control.

### Statistical analysis

All data were analysed from at least two or three independent experiments (performed in duplicate or triplicate). Statistical analyses was run on the Prism 9 software (GraphPad Software, San Diego, CA, USA) was used. If not otherwise stated, results are presented as means with standard errors.

Differences between the groups were tested with the one-way ANOVA, followed by Dunnett’s test. Statistical significance was set to p<0.05.

## RESULTS

**Figure 1:**
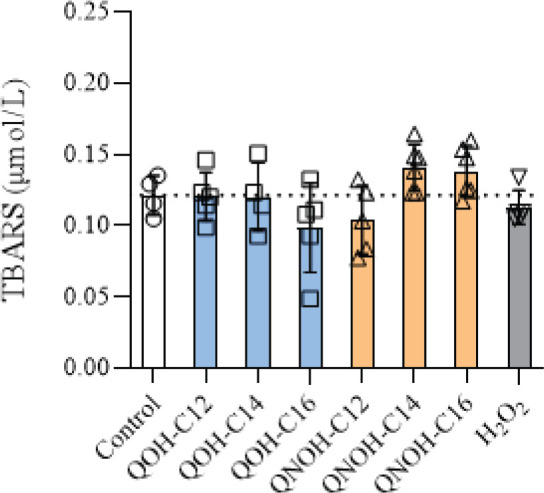
Lipid peroxidation (expressed as TBARS concentrations) in SH-SY5Y cells treated with 60 µmol/L QOH-C_12_, 6 µmol/L QOH-C_14_ or QOH-C_16_, 100 µmol/L QNOH-C_12_, and 25 µmol/L QNOH-C_14_ or QNOH-C_16_ for 4 h. Control – untreated cells; H_2_O_2_ – positive control (100 µmol/L)

**Figure 2:**
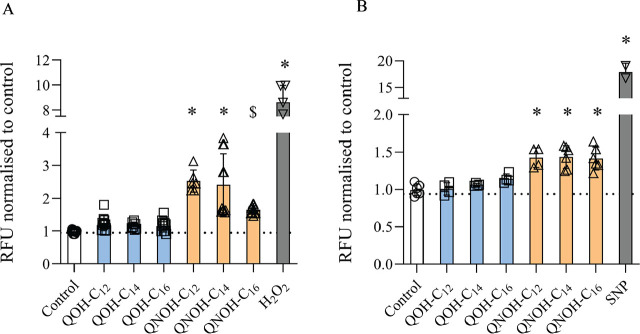
DCFDA- or DAF-FM fluorescence (expressed as RFUs normalised to control) indicating ROS (left panel) and RNS (right panel) levels in SH-SY5Y cells treated with 60 µmol/L QOH-C_12_, 6 µmol/L QOH-C_14_ or QOH-C_16_, 100 µmol/L QNOH-C_12_, and 25 µmol/L QNOH-C_14_ or QNOH-C_16_ for 4 h or 1 h, respectively. ^$^ p<0.001; ^*^p<0.0001 (Dunnett’s test). Control – untreated cells; DAF-FM – 4-amino-5-methylamino-2,7-difluorofluorescein diacetate; DCFDA – 2′,7′-dichlorofluorescein diacetate dye; H_2_O_2_ – positive control (100 µmol/L); RFU – relative fluorescence units; SNP – positive control (200 µmol/L)

None of the quinuclidine derivative treatments resulted in significant changes in TBARS concentrations in SH-SY5Y cells compared to negative control (Figure 1), but all QNOHs significantly increased ROS and RNS levels (Figure 2). Increase in ROS depended on their side chain substituent length (Figure 2A), while in RNS it remained the same, regardless of the QNOH chain length (Figure 2B).

**Figure 3:**
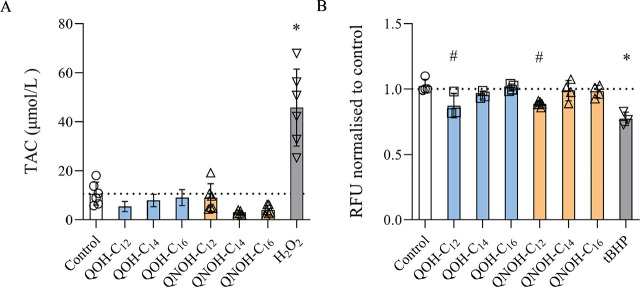
Total antioxidative capacity (TAC, expressed as µmol/L of Fe^2+^ equivalents, left panel) and MCB-fluorescence indicating GSH level (expressed as RFU normalised to control, right panel) in SH-SY5Y cells treated with 60 µmol/L QOH-C_12_, 6 µmol/L QOH-C_14_ or QOH-C_16_, 100 µmol/L QNOH-C_12_, and 25 µmol/L QNOH-C_14_ or QNOH-C_16_ for 4 h. ^#^ p<0.01; ^*^p<0.0001 (Dunnett’s test). Control – untreated cells; H_2_O_2_ or tBHP– positive controls (100 µmol/L); RFU – relative fluorescence units; SNP – positive control (200 µmol/L)

Total antioxidant capacity in cells treated with quinuclidine derivatives dropped compared to negative control, but not significantly (Figure 3A), whereas GSH levels dropped significantly only in cells treated with QNOH-C_12_ and QOH-C_12_ (Figure 3B).

**Figure 4:**
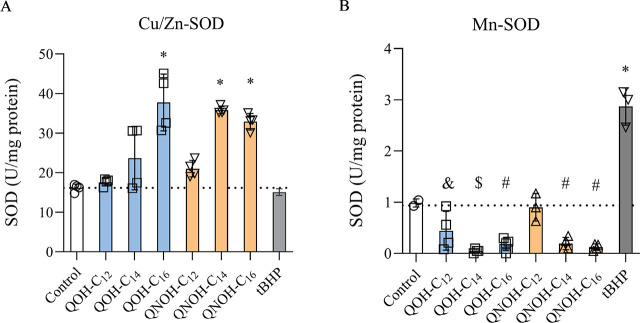
Cytosolic (Cu/Zn-SOD, left panel) and mitochondrial (Mn-SOD, right panel) superoxide dismutase activity (expressed as U/mg protein) in SH-SY5Y cells treated with 60 µmol/L QOH-C_12_, 6 µmol/L QOH-C_14_ or QOH-C_16_, 100 µmol/L QNOH-C_12_, and 25 µmol/L QNOH-C_14_ or QNOH-C_16_ for 4 h. ^&^ p<0.05; ^#^p<0.01; ^$^p<0.001; ^*^p<0.0001 (Dunnett’s test). Control – untreated cells; tBHP– positive control (100 µmol/L)

Cytosolic SOD (Cu/Zn-SOD) activity significantly increased in SH-SY5Y cells treated with QOH-C_16_, QNOH-C_14_, and QNOH-C_16_ (Figure 4A). Mitochondrial SOD (Mn-SOD) activity significantly decreased with all quinuclidine derivative treatments (Figure 4B).

**Figure 5:**
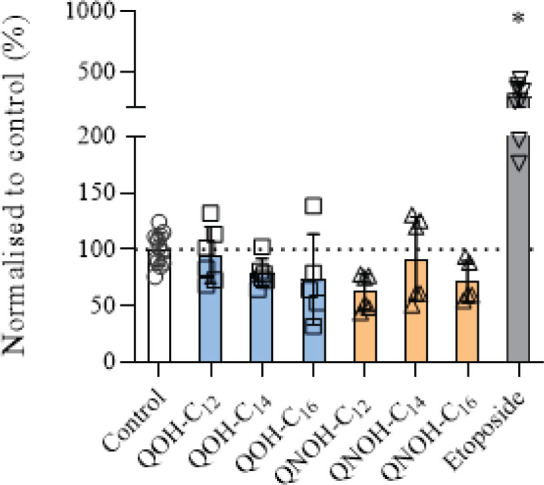
DNA damage (expressed as percentage normalised to the control) in SH-SY5Y cells treated with 60 µmol/L QOH-C_12_, 6 µmol/L QOH-C_14_ or QOH-C_16_, 100 µmol/L QNOH-C_12_, and 25 µmol/L QNOH-C_14_ or QNOH-C_16_ for 4 h. ^*^p<0.0001 (Dunnett’s test). Control – untreated cells; Etoposide – positive control (10 µmol/L)

We found no significant DNA damage in cells treated with quinuclidine derivatives (Figure 5), which may be owed to the wide distribution of results obtained with the used method.

## DISCUSSION

Our findings show varying oxidative effects relative to the structural differences between the six tested LOAEL quinuclidine derivatives in SH-SY5Y cells. QNOH compounds induced more changes than QOH compounds. ROS and RNS levels were elevated in response to QNOH treatment, regardless of the substituent alkyl chain length, and were accompanied by changes in SOD activities and to a lesser extent in GSH levels. High levels of ROS and RNS are often connected due to shared sources (such as mitochondria and NADPH oxidases) and amplification loops (ROS amplifying RNS production *via* iNOS) (28).

Cu/Zn SOD activity rose with longer alkyl chains, while Mn-SOD activity dropped significantly with all but QNOH-C_12_, which suggests an imbalance in the cell’s oxidative stress defence system, especially within the mitochondria, where Mn-SOD is critical for homeostasis (29). This sheds more light on the possible trigger for the decrease in mitochondrial membrane potential (ΔΨm) and on the induction of mitochondria-mediated apoptosis observed in our earlier study (14). In addition, increased cytosolic Cu/Zn SOD may reflect a defensive mechanism to neutralise ROS/RNS spilling over from stressed mitochondria into the cytosol (29). On the other hand, this increase may be owed to general oxidative stress but cannot compensate for lower Mn-SOD activity in mitochondria and the resulting mitochondrial damage.

The finding that only C_12_ derivatives led to GSH depletion suggests that the substituent alkyl chain length influences how these compounds interact with cellular defence mechanisms. Although GSH levels in SH-SY5Y cells dropped following the treatment with QOH-C_12_ and QNOH-C_12_, a reduction in total antioxidant capacity (TAC) is evident across all samples, indicating that all tested quinuclidine derivatives increase oxidative stress and cause redox imbalance, as suggested by Micheli et al. (30) in their docetaxel study in SH-SY5Y cells.

The induction of ROS/RNS in our study without lipid peroxidation (no significant changes in TBARS) suggests that ROS/RNS generation is primarily triggered by cellular signalling (e.g. peroxides) rather than the highly reactive species like hydroxyl radicals or superoxide anions that directly attack lipids (31). This is a common feature of many synthetic compounds that disrupt mitochondrial function or electron transport chains (28). Interestingly, treatment with H_2_O_2_ also did not induce significant lipid peroxidation in SH-SY5Y cells, suggesting that either the 100 µmol/L concentration was insufficient to overcome cellular antioxidant defences or that additional pro-oxidant conditions (e.g., transition of metal ions) are required to trigger lipid peroxidation.

Moreover, the lack of significant DNA damage despite ROS/RNS generation suggests that the compounds may not directly cause DNA breaks. However, the potential for indirect DNA damage may still exist, as these effects are not always immediately detected, depending on experimental conditions (33, 34). As far as we know, the processes triggered in our study could end in cell death in 24 h, as some compounds induce cumulative cellular damage over time, especially through mechanisms like oxidative stress, mitochondrial dysfunction, and enzyme inhibition (35,36,37).

Generally, the tested quinuclidine compounds induced oxidative stress with a potential to modify key antioxidant enzyme activities by affecting the mitochondrial function. This aligns with earlier reports of mitochondrial dysfunction caused by similar classes of compound (38,39,40).

Judging by what we know about the tested compounds at this point, the safe dose for each needs to be lower than the LOAEL used in this study: 60 µmol/L for QOH-C_12_, 6 µmol/L for QOH-C_14_ and QOH-C_16_, 100 µmol/L for QNOH-C_12_, and 25 µmol/L for QNOH-C_14_ and QNOH-C_16_. Considering that QOH-C_12_ inhibits cholinesterase (ChE) activity in the range of 9–13 µmol/L (14), it has potential for further development, as its LOAEL is about six times higher than its inhibition constant (K_i_). Even more promising is QNOH-C_12_, whose ChE inhibition window of 5–14 µmol/L (14), and the 100 µmol/L LOAEL provides a nearly 10-fold safety margin. However, the inhibition potencies for QOH-C_14_, QOH-C_16_, QNOH-C_14_, and QNOH-C_16_ leave no room for further development as ChE inhibitors, as they are in the same range as their LOAEL (14).

All these findings raise questions to be addressed by further investigation, especially in terms of how chain length and functional groups on the quinuclidine core modulate oxidative stress responses and affect mitochondrial integrity.

## CONCLUSION

*N*-alkyl quaternary quinuclidines (Q(N)OHs) in our study induced oxidative stress in SH-SY5Y cells primarily through ROS and RNS but did not cause significant lipid peroxidation or DNA damage. Although the activation of the non-enzymatic antioxidative defence system (evidenced by insignificant GSH depletion) was modest, the cellular response of the enzymatic antioxidative defence system (cytosolic Cu/Zn-SOD) was highly active, which confirms oxidative stress. Moreover, the observed decrease in mitochondrial Mn-SOD activity suggests mitochondrial dysfunction.

These findings highlight the potential of Q(N)OHs to disrupt cellular redox homeostasis, with implications for further studies on their safety and therapeutic potential, including broader cellular effects and dose optimisation.
